# Development and validation of nomograms for predicting overall survival and cancer specific survival in locally advanced breast cancer patients: A SEER population-based study

**DOI:** 10.3389/fpubh.2022.969030

**Published:** 2022-09-20

**Authors:** Fangxu Yin, Song Wang, Chong Hou, Yiyuan Zhang, Zhenlin Yang, Xiaohong Wang

**Affiliations:** ^1^Department of Thyroid and Breast Surgery, Binzhou Medical University Hospital, Binzhou, China; ^2^Department of Gastroenterology, Yantai Affiliated Hospital of Binzhou Medical University, Yantai, China; ^3^Department of Reproductive Endocrinology, Affiliated Reproductive Hospital of Shandong University, Jinan, China

**Keywords:** locally advanced breast cancer, overall survival, prognosis, cancer specific survival, SEER, nomogram

## Abstract

**Background:**

For patients with locally advanced breast cancer (LABC), conventional TNM staging is not accurate in predicting survival outcomes. The aim of this study was to develop two accurate survival prediction models to guide clinical decision making.

**Methods:**

A retrospective analysis of 22,842 LABC patients was performed from 2010 to 2015 using the Surveillance, Epidemiology and End Results (SEER) database. An additional cohort of 200 patients from the Binzhou Medical University Hospital (BMUH) was analyzed. The least absolute shrinkage and selection operator (LASSO) regression was used to screen for variables. The identified variables were used to build a survival prediction model. The performance of the nomogram models was assessed based on the concordance index (C-index), calibration plot, receiver operating characteristic (ROC) curve, and decision curve analysis (DCA).

**Results:**

The LASSO analysis identified 9 variables in patients with LABC, including age, marital status, Grade, histological type, T-stage, N-stage, surgery, radiotherapy, and chemotherapy. In the training cohort, the C-index of the nomogram in predicting the overall survival (OS) was 0.767 [95% confidence intervals (95% CI): 0.751–0.775], cancer specific survival (CSS) was 0.765 (95% CI: 0.756–0.774). In the external validation cohort, the C-index of the nomogram in predicting the OS was 0.858 (95% CI: 0.812–0.904), the CSS was 0.866 (95% CI: 0.817–0.915). In the training cohort, the area under the receiver operator characteristics curve (AUC) values of the nomogram in prediction of the 1, 3, and 5-year OS were 0.836 (95% CI: 0.821–0.851), 0.769 (95% CI: 0.759–0.780), and 0.750 (95% CI: 0.738–0.762), respectively. The AUC values for prediction of the 1, 3, and 5-year CSS were 0.829 (95% CI: 0.811–0.847), 0.769 (95% CI: 0.757–0.780), and 0.745 (95% CI: 0.732–0.758), respectively. Results of the C-index, ROC curve, and DCA demonstrated that the nomogram was more accurate in predicting the OS and CSS of patients compared with conventional TNM staging.

**Conclusion:**

Two prediction models were developed and validated in this study which provided more accurate prediction of the OS and CSS in LABC patients than the TNM staging. The constructed models can be used for predicting survival outcomes and guide treatment plans for LABC patients.

## Introduction

Breast cancer is the most common malignancy in women worldwide. About 1.2 million women have been diagnosed with breast cancer, and 500,000 women die from breast cancer yearly worldwide (1). Locally advanced breast cancer (LABC) is a heterogeneous entity that includes stage IIB (T3N0M0) and IIIA primary breast cancers which are treated with radical surgery. Cancers involving the skin, chest wall and lymph nodes are not treatable with radical surgery. Stage IIIB and IIIC breast cancers are associated with extensive lymph node involvement (2). For over 100 years, radical surgery has been considered the standard treatment for many types of breast cancers, including LABC (3). Haagensen and Stout showed that the 5-year recurrence rate for LABC treated with radical surgery alone was 46%, with a 5-year survival rate of 6% (4). Although radiotherapy has been applied in patients with inoperable LABC (2), the outcomes are poor. It has been reported that radiotherapy combined with surgery is superior to any monotherapy in treating LABC. Currently, the prognosis of LABC patients is poor regardless of the treatment given. Hortobagyi evaluated 9,055 patients with stage III breast cancer treated with surgery plus radiotherapy. Results showed that the 5-year and 10-year survival rates of patients were 33 and 22%, respectively (5). In 1970's, neoadjuvant chemotherapy (preoperative chemotherapy) was the main treatment for LABC with remarkable success (6). In a study by Broadwater et al., postoperative complications associated with preoperative chemotherapy and postoperative adjuvant chemotherapy were evaluated in 200 LABC patients. It was found that patients treated with preoperative chemotherapy had no significant postoperative complications. Among the complications identified were subcutaneous effusion and poor healing of the surgical incision (7).

Several factors affect the prognosis of LABC patients, including intrinsic characteristics of the tumor [molecular typing (8, 9), local staging (10), lymph node status (11), histopathological grading (12, 13), tumor location (14, 15)], and patient's factors such as [age (16, 17), gender, menstrual status, and treatment plan (18–21)]. Moreover, the choice of therapy depends on the type of breast cancer, biological indicators, molecular typing of breast cancer, and physical condition of the patient. This makes it difficult to make treatment decisions for LABC patients. Therefore, accurate risk stratification of LABC disease is essential for proper prognostic assessment and treatment selection. Currently, the American Joint Committee on Cancer (AJCC) 7th edition TNM staging is the most widely used prognostic assessment tool for LABC (11). However, this tool cannot accurately predict the survival of LABC patients. Therefore, nomograms, which integrate multiple factors to quantify the likelihood of clinical events, have been widely adopted in clinical practice (22, 23). Nomograms are more accurate in evaluating clinical events compared with traditional TNM staging (24). However, no study has established a nomogram for predicting the survival of LABC patients. This study aimed to establish and validate two nomograms for assessing LABC patients based on the SEER database using multiple independent prognostic factors.

## Materials and methods

### Data sources and patient selection

The SEER database is a large clinical database funded by the National Cancer Institute (NCI), which contains data concerning cancer incidence and survival from 18 cancer registries covering approximately 30% of the US population and is updated annually. A data-use agreement was reached to allow us access the data, with the username, 17,844-Nov2020. The Ethics Committee of BMUH approved this study, and the requirement for informed consent was waived because patient information was anonymized at every step of the study, including during data collation and statistical analysis.

We collected data of patients with locally advanced breast cancer registered in the Surveillance, Epidemiology and End Results (SEER) database from 2010 to 2015 and from the medical record system of the BMUH. Inclusion criteria were as follows: (i) the age of diagnosis was between 2010 and 2015; (ii) information on patients with stage IIB(T3N0M0)/IIIA/IIIB/IIIC according to the 7th edition of the AJCC system; (iii) no other confirmed tumor except for patients with locally advanced invasive ductal carcinoma (ICD-O-3:8500/3); (iv) complete clinical and pathological data; and (v) complete follow-up information. Exclusion criteria were: (i) patient age <18 years at diagnosis; (ii) unknown data of race, grade, surgery, marital status; (iii) the follow-up time was 0 and unknown; and (iv) patients with Tx stages screened according to the 7th edition AJCC staging. Finally, 22,842 LABC patients were screened. If the sample size of the missing value in the database was <5% of the total number of people, it will be deleted. The OS was defined as the time interval from the date of diagnosis to the date of death or the last follow-up due to any cause, and CSS was defined as the time interval from the date of diagnosis to the date of death or the last follow-up of LABC.

Clinical data of 200 patients with invasive ductal carcinoma of the breast at BMUH were retrospectively analyzed, and we obtained clinicopathological parameters of the patients, including age, race, sex, laterality, marital status, grade, TNM stage, subtype, surgery, radiotherapy, and chemotherapy, from 2010 to 2015. Inclusion criteria were: (i) age≥18 years, surgery was primary; (ii) tumor was primary, pathological diagnosis was invasive ductal carcinoma of the breast, pathological diagnosis was based on the primary site and according to the ICD-O-3; (iii) information of patients with clinical stage IIB (T3N0M0)/IIIA/IIB/IIIC; (iv) complete clinical data and postoperative follow-up data. Exclusion criteria were: (i) patients were <18 years old at diagnosis; (ii) data on race, grade, surgery, and marital status were not available; (iii) follow-up time was 0 and not available; and (iv) patients with Tx stage screened according to the 7th edition AJCC staging. In this study, the starting point of follow-up was the diagnosis of invasive ductal carcinoma with LABC, with OS as the primary endpoint and CSS as the secondary endpoint. A follow-up was conducted through direct contact with patients or by telephone conversation with patients. The follow-up ended on December 31, 2021.

### Construction and validation of the nomogram

The study was designed based on transparent reporting of a multivariable prediction model for individual prognosis or diagnosis (TRIPOD) (25). The following data were obtained from the SEER database: age at diagnosis, marital status, sex, laterality, race, grade, subtype, T-stage, N-stage, surgery, radiotherapy, and chemotherapy. Approximately 70% (*n* = 15,917) of LABC patients were assigned to the training cohort and the remaining 30% (*n* = 6,925) to the validation cohort. Then LASSO regression analysis was performed to select the best predictors of the 1-year, 3-year and 5-year survival in the training cohort. Before construction of the Cox proportional risk model, all included variables meet the proportional risk assumption. The independent prognostic factors were integrated and included in the nomogram after identification to assess the OS and CSS of LABC patients at 1, 3, and 5 years. Finally, the prediction performance of the nomogram was evaluated based on the concordance C-index and ROC curve. Calibration curve analysis were used to evaluate the accuracy of the nomogram. DCA was used to evaluate the net benefit of the nomogram. The model was identified and calibrated internally and externally.

### Risk stratification based on the nomogram

The selected variables were scored according to the adjusted β-regression coefficients in the multiple regression in the risk model. The total risk score of each LABC patient was obtained, and then the median of the total scores of all patients was used as the critical value, with those above the critical value being the high-risk group and the rest being the low-risk group. Kaplan-Meier curve was then used to evaluate the OS and CSS of LABC patients.

### Statistical analysis

IBM SPSS Statistics Software, version 22.0 and R Statistical Software, version 4.1.2 were used for statistical analysis. SPSS software was used to compare the survival prognosis of LABC patients in the training cohort *via* the log-rank method analysis. Kaplan-Meier survival curve analysis was used to evaluate the correlation of multiple variables with OS and CSS. First, the R-language random sampling function was used to randomly divide the patients into Derivation and validation cohort. SPSS software was used to assess the clinicopathological data differences between the training and validation cohorts. Lasso regression analysis was used to assess the factors affecting the survival prognosis of patients in the training cohort. After calculating the variance expansion factor (VIF), the Vif values of each covariate are <4, suggesting that the multicollinearity between variables is not significant. The product of each covariate was added in turn as an interaction term, which was included in the Cox regression model for analysis, and no obvious interaction effect was found. The variables screened in LASSO regression analysis were included in Cox proportional hazards model. R software was used to build the nomogram, plot the calibration curves, ROC curves, and DCA curves, and to calculate the C-index. The calibration curves were plotted using the Bootstrap method. The validation cohort was repeatedly sampled 1,000 times for internal validation. In the calibration curve, the closer the curve is to the ideal 45°reference line, the closer the predicted value is to the actual observed value. The ROC curve is based on a series of different dichotomous approaches (cut-off values or decision thresholds) with the true positive rate (sensitivity) as the vertical coordinate and the false positive rate (1-specificity) as the horizontal coordinate. AUC is defined as the area under the ROC curve. The C-index was used to evaluate the predictive value of the nomogram. The maximum and minimum values of the C-index are 1 (indicating 100% prediction accuracy) and 0.5, respectively. A C-index closer to 1 indicates better accuracy of the prediction model. DCA was used to compare the clinical benefits of the nomogram and the AJCC staging system. The threshold probability represented the horizontal coordinate of the curve, whereas the net benefit rate after subtracting the benefit from the harm represented the vertical coordinate. The patient's risk probability of a temporal event was presented as Pi when various evaluation methods reached a certain value. Pi is defined as positive when it reaches a certain threshold value (noted as Pt). A model with a curve close to two reference lines indicated that the model has no value, while that with a curve above the reference line in a large threshold interval indicated a better model. Finally, the patients were divided into two strata based on prediction scores. Kaplan-Meier survival curves were used to describe the differences and associations between the two strata. Log-rank tests were conducted to evaluate the effects of each variable on OS and CSS. All statistical tests were two-sided, and *P* < 0.05 were considered statistically significant.

## Results

### Data sources and patient selection

**Figure 1:**
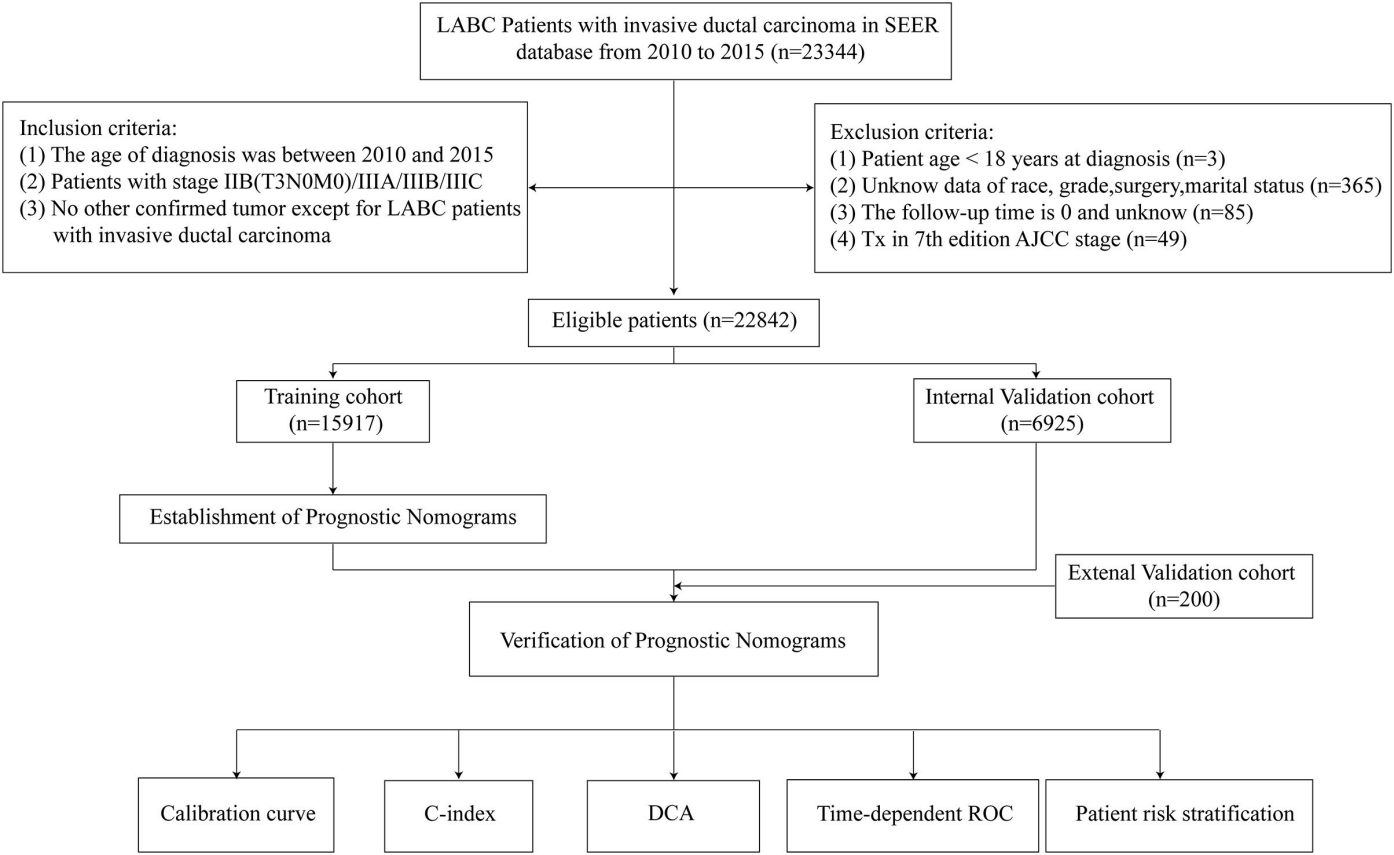
Flowchart of participant inclusion and exclusion.

Demographic data of 22,842 eligible LABC patients were collected from the SEER database (15,917 in the training cohort and 6,925 in the validation cohort) and 200 eligible LABC patients of external verification from the BMUH. Screening protocols and demographic characteristics are shown in Figure 1 and Table 1, respectively. There was no difference in demographic characteristics between training cohort and internal validation cohort (*P* > 0.05).

**Table 1:** Characteristics of patients with LABC in the training and validation group.

**Characteristics**	**Training cohort**	**Internal validation cohort**	**Overall**	**External validation cohort**	**T vs. IV**	**T vs. EV**
	**(*n* = 15,917)**	**(*n* = 6,925)**	**(*n* = 22,842)**	**(*n* = 200)**	***P***	***P***
	**No. of patients (%)**	**No. of patients (%)**	**No. of patients (%)**	**No. of patients (%)**		
**Age**					0.11	0.46
<40	1,665 (10.5)	762 (11.0)	2,427 (10.6)	19 (9.5)		
40–59	7,776 (48.8)	3,317 (47.9)	11,093 (48.6)	100 (50.0)		
60–79	5,235 (32.9)	2,252 (32.5)	7,487 (32.8)	60 (30.0)		
≧80	1,241 (7.8)	594 (8.6)	1,835 (8.0)	21 (10.5)		
**Race**					0.74	<0.01
White	11,778 (74.0)	5,103 (73.7)	16,881 (73.9)	0 (0.0)		
Black	2,522 (15.8)	1,125 (16.2)	3,647 (16.0)	0 (0.0)		
Others^a^	1,617 (10.2)	697 (10.1)	2,314 (10.1)	200 (100.0)		
**Sex**					0.14	0.52
Female	15,717 (98.7)	6,854 (99.0)	22,571 (98.8)	199 (99.5)		
male	200 (1.3)	71 (1.0)	271 (1.2)	1 (0.5)		
**Laterality**					0.28	0.73
Left	8,043 (50.5)	3,489 (50.4)	11,532 (50.5)	100 (50.0)		
Right	7,871 (49.5)	3,435 (49.6)	11,306 (49.5)	100 (50.0)		
Bilateral	3 (0.0)	1 (0.0)	4 (0.0)	0 (0.0)		
**Marital status**					0.43	0.70
Married	8,478 (53.3)	3,675 (53.1)	12,153 (53.2)	101 (50.5)		
Single	3,331 (20.9)	1,410 (20.4)	4,813 (21.1)	46 (23.0)		
Others^b^	4,108 (25.8)	1,840 (26.5)	5,876 (25.7)	53 (26.5)		
**Grade**					0.46	0.33
I	905 (5.7)	375 (5.4)	1,280 (5.6)	6 (3.0)		
II	5,571 (35.0)	2445 (35.3)	8,016 (35.1)	76 (38.0)		
III	9,367 (58.8)	4,071 (58.8)	13,438 (58.8)	118 (59.0)		
IV	74 (0.5)	34 (0.5)	108 (0.5)	0 (0.0)		
**AJCC T**					0.13	0.57
T0-1	1,982 (12.5)	797 (11.6)	2,779 (12.1)	27 (13.5)		
T2	4,771 (30.0)	2,128 (30.7)	6,899 (30.2)	62 (31.0)		
T3	5,991 (37.6)	2,655 (38.3)	8,646 (37.9)	66 (33.0)		
T4	3,173 (19.9)	1,345 (19.4)	4,518 (19.8)	45 (22.5)		
**AJCC N**					0.71	0.28
N0	2,367 (14.9)	1,062 (15.3)	3,429 (15.0)	29 (14.5)		
N1	3,975 (25.0)	1,733 (25.0)	5,708 (25.0)	43 (21.5)		
N2	6,358 (39.9)	2,767 (40.0)	9,125 (39.9)	93 (46.5)		
N3	3,217 (20.2)	1,363 (19.7)	4,580 (20.1)	35 (17.5)		
**Subtype**					0.33	0.83
Luminal A	8,810 (55.3)	3,856 (55.6)	12,666 (55.4)	115 (57.5)		
Luminal B	2,606 (16.4)	1,129 (16.3)	3,735 (16.4)	32 (16.0)		
Her-2	1,575 (9.9)	634 (9.2)	2,209 (9.7)	16 (8.0)		
Triple-negative	2,926 (18.4)	1,306 (18.9)	4,232 (18.5)	37 (18.5)		
**Surgery**					0.23	<0.01
BCS	3,519 (22.1)	1,602 (23.1)	5,121 (22.4)	67 (33.5)		
Mastectomy	11,100 (69.7)	4,761 (68.8)	15,861 (69.5)	123 (61.5)		
No surgery	1,298 (8.2)	562 (8.1)	1,860 (8.1)	10 (5.0)		
**Radiation**					0.08	0.41
Yes	9,343 (58.7)	4,185 (60.4)	13,528 (59.2)	112 (56.0)		
No/Unknown	6,531 (41.3)	2,779 (39.6)	9,314 (40.8)	88 (44.0)		
**Chemotherapy**					0.65	0.33
Yes	12,848 (80.7)	5,572 (80.5)	18,420 (80.6)	156 (78.0)		
No/Unknown	3,069 (19.3)	1,353 (19.5)	4,422 (19.4)	44 (22.0)		

BCS, breast-conserving surgery; HR, hazard ratio; 95 CI, 95% confidence interval; Others^a^, including Asian or Pacific Islander and American Indian/Alaska Native; Others^b^, including separated, divorced and widowed; T, Training cohort; IV, Internal validation cohort; EV, External validation cohort.

In the SEER database, about half of the patients were older than 60 years (40.8%), 73.9% were white, and 16.0% were black and other races were 10.1%. Most patients were females (98.8%) and had poorly differentiated tumors (grade II/III). The TNM staging indicated that the distribution of patients was relatively even, with 37.9% of patients in the T3 stage and 39.9% in the N2 stage. Molecular staging showed that 55.4% of the patients had Luminal A, 16.4% had Luminal B, 9.7% had Her-2-positive, and 18.5% had triple-negative breast cancer. Moreover, 8.1% of the patients did not undergo surgery, 40.8% did not undergo radiotherapy, and 19.4% did not undergo chemotherapy. In the external validation cohort, 40.5% of patients were over 60 years of age, and all patients were Asian. Female patients accounted for 99.5% of patients and had poorly differentiated tumors (grade II/III). TNM staging showed a more even distribution as well, with 33.0% of patients in stage T3 and 46.5% in stage N2. Molecular staging showed that 57.5% of patients were Luminal A, 16.0% were Luminal B, 8.0% were Her-2-positive, and 18.5% had triple-negative breast cancer. In addition, 5.0% of patients did not undergo surgery, 44.0% did not receive radiotherapy, and 22.0% did not receive chemotherapy.

### Establishment and validation of prognostic nomograms

**Figure 2:**
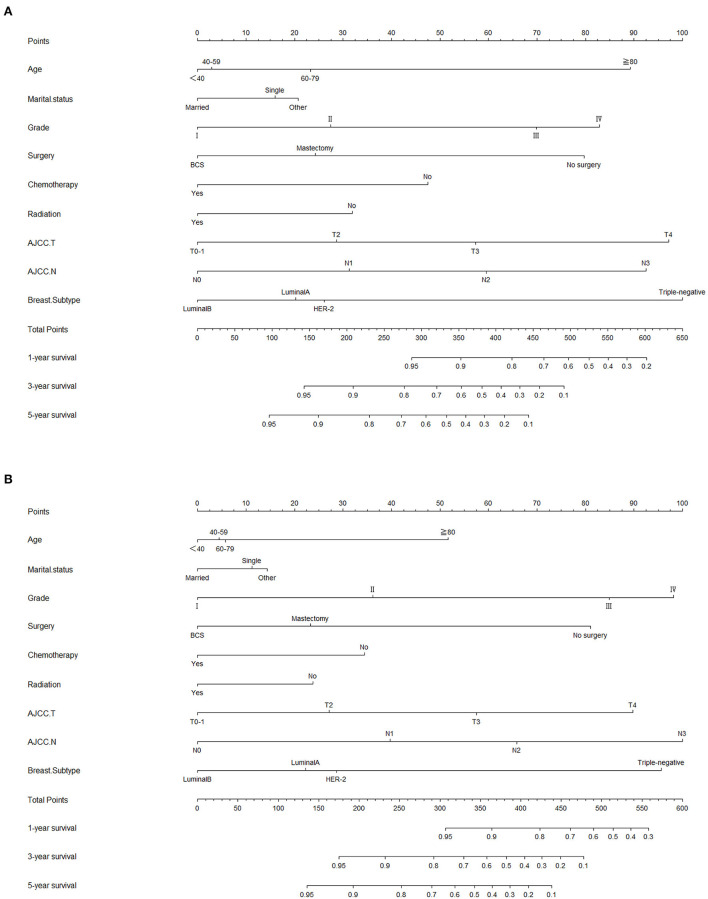
Nomograms for predicting 1-, 3-, and 5-year **(A)** OS and **(B)** CSS of patients with LABC.

**Figure 3:**
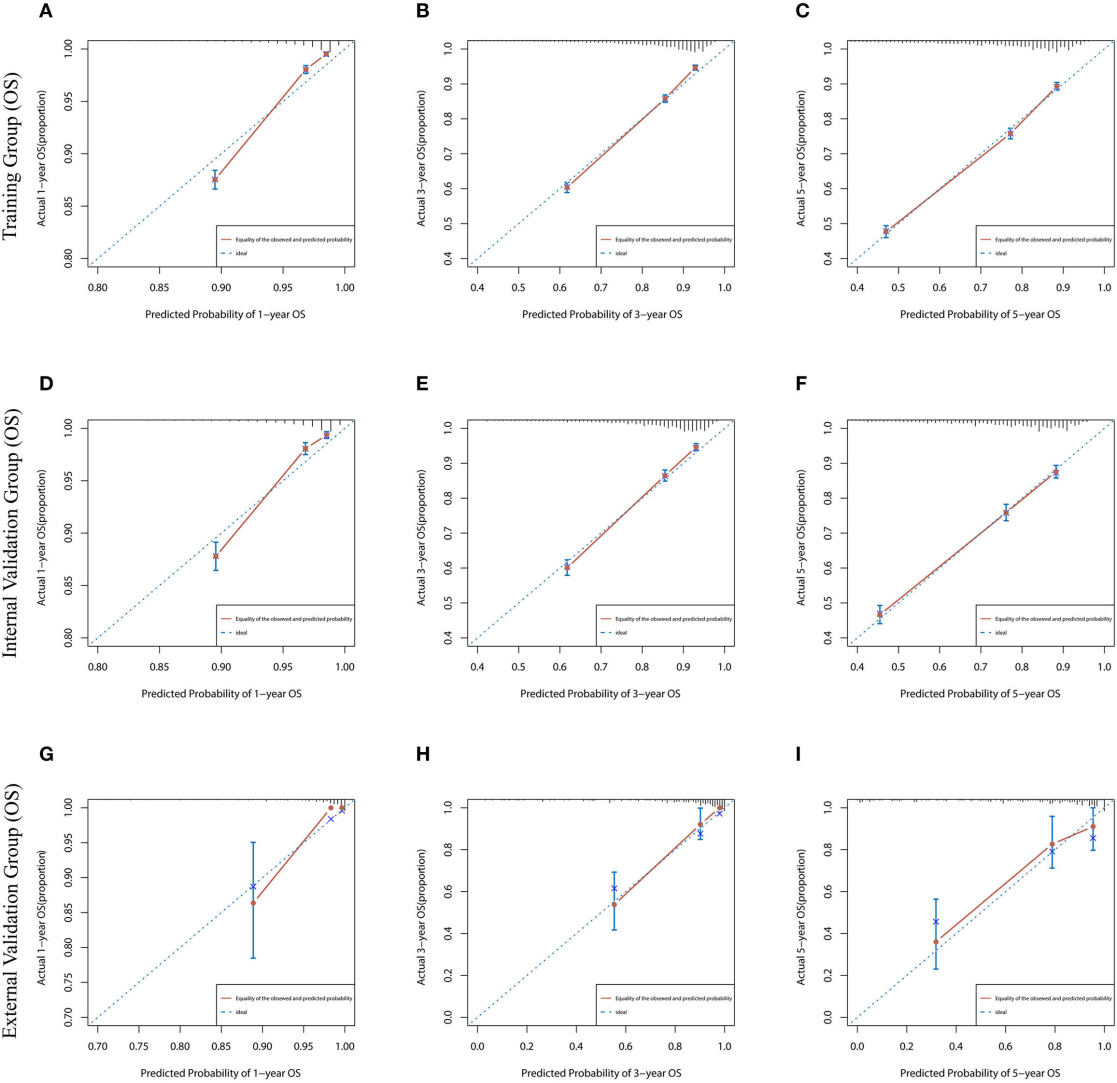
The calibration curves for predicting OS at **(A)** 1-year and **(B)** 3-year and **(C)** 5-year in the training cohort, and at **(D)** 1-year **(E)** 3-year and **(F)** 5-year in the internal validation cohort, and at **(G)** 1-year **(H)** 3-year and **(I)** 5-year in the external validation cohort.

**Figure 4:**
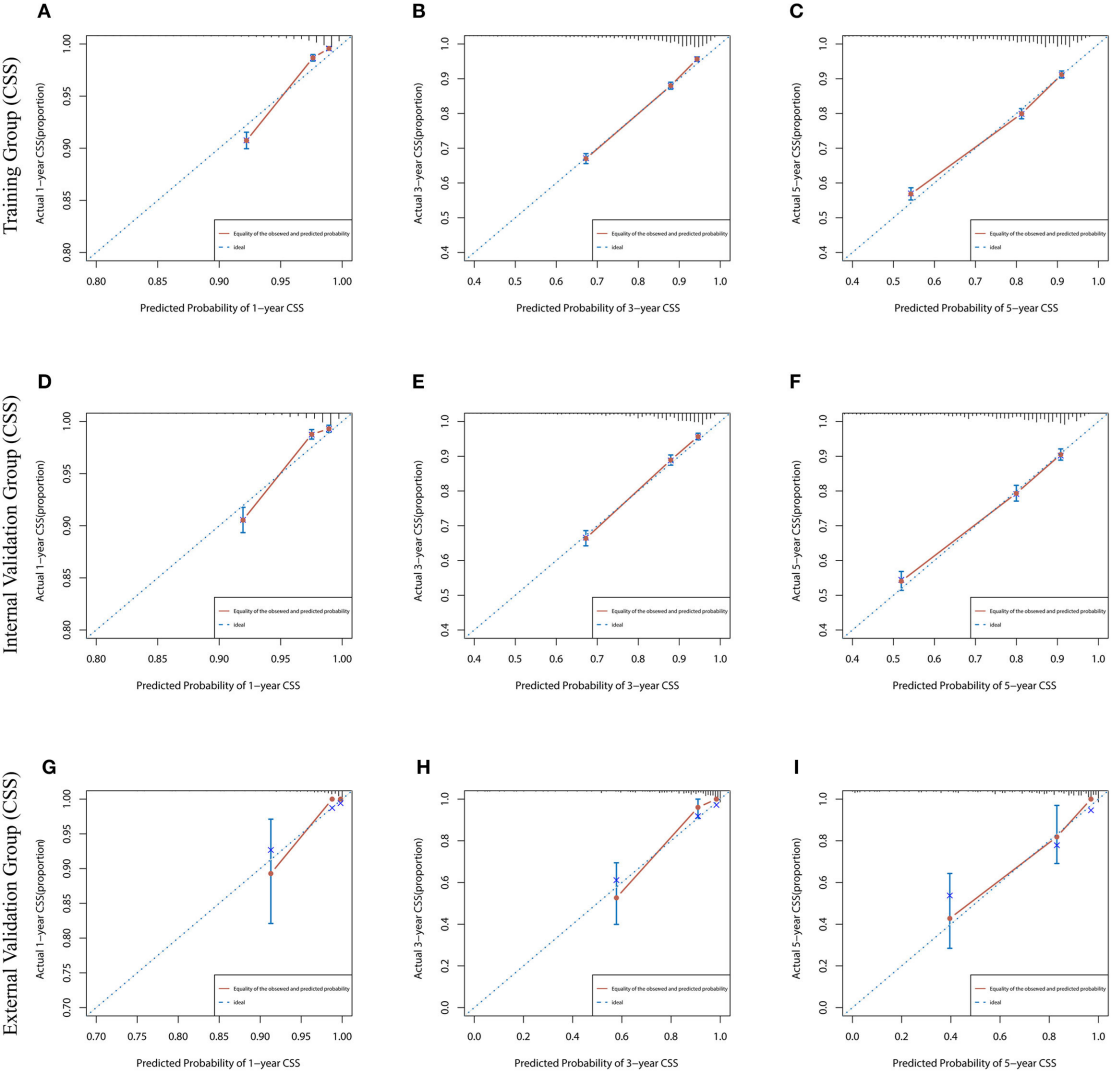
The calibration curves for predicting CSS at **(A)** 1-year and **(B)** 3-year and **(C)** 5-year in the training cohort, and at **(D)** 1-year **(E)** 3-year and **(F)** 5-year in the internal validation cohort, and at **(G)** 1-year **(H)** 3-year and **(I)** 5-year in the external validation cohort.

**Figure 5:**
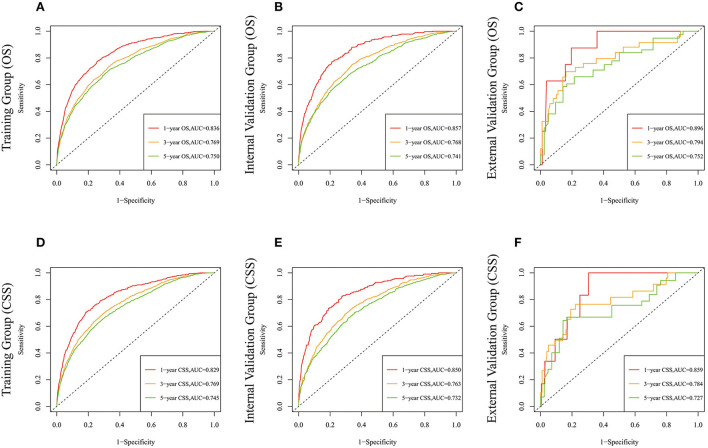
The time-dependent ROC curves of the nomogram predicting OS at **(A)** 1-year and 3-year and 5-year in the training cohort, and at **(B)** 1-year 3-year and 5-year in the internal validation cohort, **(C)** 1-year and 3-year and 5-year in the external training cohort. The time-dependent ROC curves of the nomogram predicting CSS at **(D)** 1-year and 3-year and 5-year in the training cohort, and at **(E)** 1-year 3-year and 5-year in the internal validation cohort, **(F)** 1-year and 3-year and 5-year in the external training cohort.

**Figure 6:**
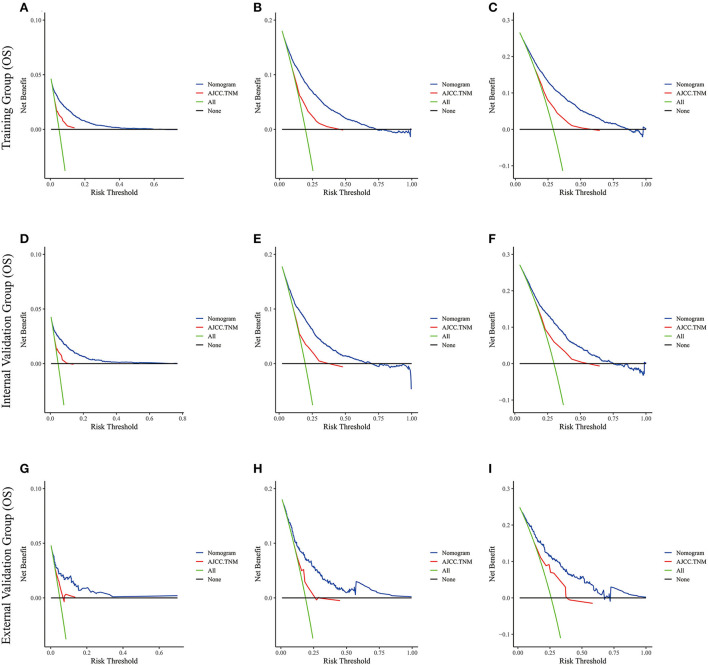
The decision curve analysis of the nomogram and AJCC.TNM for OS at **(A)** 1-year and **(B)** 3-year and **(C)** 5-year in the training cohort, and at **(D)** 1-year **(E)** 3-year and **(F)** 5-year in the internal validation cohort, and at **(G)** 1-year **(H)** 3-year and **(I)** 5-year in the external validation cohort.

**Figure 7:**
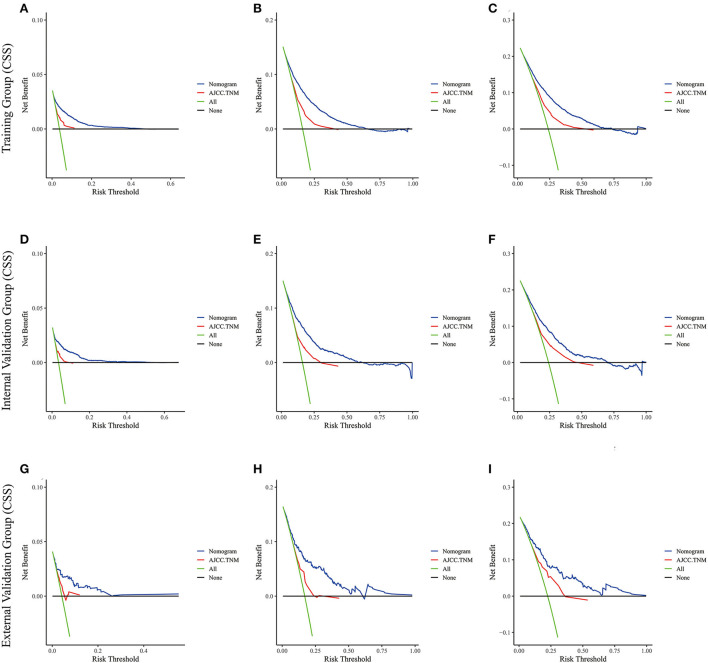
The decision curve analysis of the nomogram and AJCC.TNM for CSS at **(A)** 1-year and **(B)** 3-year and **(C)** 5-year in the training cohort, and at **(D)** 1-year **(E)** 3-year and **(F)** 5-year in the internal validation cohort, and at **(G)** 1-year **(H)** 3-year and **(I)** 5-year in the external validation cohort.

Lasso regression filters out 9 variables, including age (OS coefficient: 0.321; CSS coefficient: 0.148), marital status (OS coefficient: 0.129; CSS coefficient: 0.100), grade (OS coefficient: 0.340; CSS coefficient: 0.462), histological type (OS coefficient: 0.273; CSS coefficient: 0.292), T-stage (OS coefficient: 0.327; CSS coefficient: 0.340), N-stage (OS coefficient: 0.279; CSS coefficient: 0.327), surgery (OS coefficient: 0.414; CSS coefficient: 0.477), radiotherapy (OS coefficient: 0.313; CSS coefficient: 0.269), and chemotherapy (OS coefficient: 0.626; CSS coefficient: 0.519) (Supplementary Figure 1). All variables passed the proportional risk hypothesis test. The variables were integrated to create two nomograms for OS and CSS prediction at 1, 3, and 5 years for LABC patients (Figure 2). The scores of each independent prognostic factor were summed to obtain a total score before estimating the probabilities of OS and CSS at 1, 3, and 5 years. The C-index and calibration curves were used to validate the training and validation cohorts. The nomograms obtained a superior C-index compared with the AJCC staging system [OS: 0.767 (95% CI, 0.751–0.775) in the training cohort; 0.765 (95% CI, 0.753–0.777) in the internal validation cohort; 0.858 (95% CI, 0.812–0.904) in the external validation cohort]; [CSS: 0.765 (95% CI, 0.756–0.774) in the training cohort; 0.762 (95% CI, 0.748–0.776) in the internal validation cohort; 0.866 (95% CI, 0.817–0.915) in the external validation cohort] (Supplementary Table 1). The calibration curves showed good agreement between the survival probabilities predicted by the nomogram and the actual observations in both the training and validation cohorts (Figures 3, 4). ROC curves were used to evaluate the discriminatory ability of the nomograms, Figures 5A–C illustrates the 1-, 3-, and 5-year values of the AUC regarding the nomogram for OS [training cohort: 1-year OS 0.836 (95% CI, 0.821–0.851); 3-year OS 0.769 (95% CI, 0.759–0.780); 5-year OS 0.750 (95% CI, 0.738–0.762); internal validation cohort: 1-year OS 0.857 (95% CI, 0.836–0.877); 3-year OS 0.768 (95% CI, 0.752–0.784); 5-year OS 0.741 (95% CI, 0.722–0.760); external validation cohort: 1-year OS 0.896 (95% CI, 0.810–0.981); 3-year OS 0.794 (95% CI, 0.685–0.903); 5-year OS 0.752 (95% CI, 0.639–0.865)] and Figures 5D–F illustrates the 1-, 3-, and 5-year values of the AUC regarding the nomogram for CSS [training cohort: 1-year CSS 0.829 (95% CI, 0.811–0.847); 3-year CSS 0.769 (95% CI, 0.757–0.780); 5-year CSS 0.745 (95% CI, 0.732–0.758); validation cohort: 1-year CSS 0.850 (95% CI, 0.823–0.876); 3-year CSS 0.763 (95% CI, 0.745–0.781); 5-year CSS 0.732 (95% CI, 0.712–0.752); external validation cohort: 1-year CSS 0.859 (95% CI, 0.764–0.955); 3-year CSS 0.784 (95% CI, 0.668–0.899); 5-year CSS 0.727 (95% CI, 0.595–0.860)] (Supplementary Table 2). The DCA for each prediction model and the AJCC 7th edition TNM staging are shown in Figures 6, 7 for OS and CSS. The superior net benefit indicated that the nomogram showed more accurate values than the AJCC 7th edition TNM staging.

### Risk stratification

**Figure 8:**
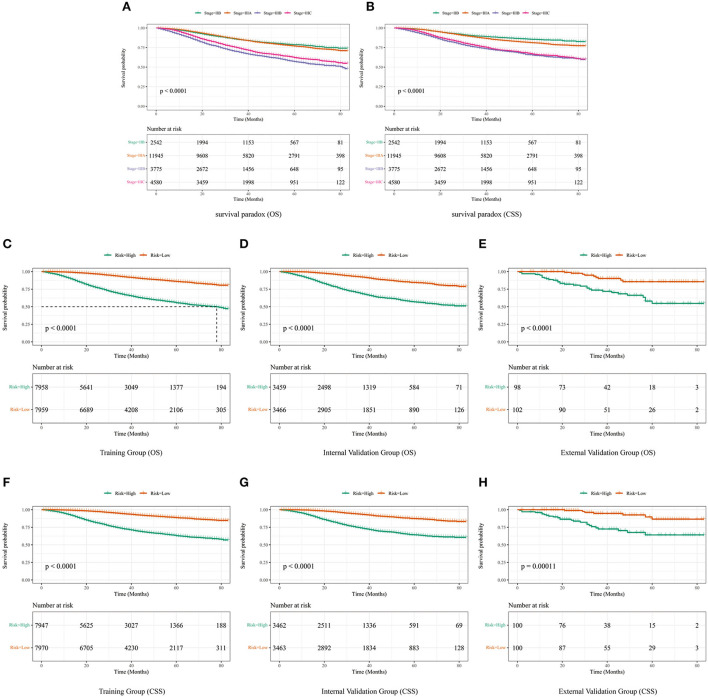
The difference in OS **(A)** and CSS **(B)** among IIB, IIIA, IIIB, and IIIC patients. **(C)** OS in the subgroups according to the risk stratification in the training cohort. **(D)** OS in the subgroups according to the risk stratification in the internal validation cohort. **(E)** OS in the subgroups according to the risk stratification in the external validation cohort. **(F)** CSS in the subgroups according to the risk stratification in the training cohort. **(G)** CSS in the subgroups according to the risk stratification in the internal validation cohort. **(H)** CSS in the subgroups according to the risk stratification in the external validation cohort.

Although it is widely used to evaluate the prognosis of various tumors, the AJCC staging system produces a survival paradox for LABC. We found that patients with stage IIIB had worse survival outcomes than those with stage IIIC (Figures 8A,B). The median risk scores were: [(OS: training set, 10.799; internal validation set, 0.937; external validation set, 0.885); (CSS: training set, 15.318; internal validation set, 0.959; external validation set, 0.886)]. LABC patients with total risk scores above the median were defined as the high-risk group, and the rest were defined as the low-risk group. Kaplan-Meier curves were then used to assess OS and CSS in LABC patients (Figures 8C–H). For the training cohort: the incidence of 1, 3, and 5-year OS was 99.1, 92.7, and 86.0% in the low-risk group and 91.7, 68.4, and 55.5% in the high-risk group, respectively; the incidence of 1-, 3-, and 5-year CSS was 99.3, 94.3, and 88.6% in the low-risk group and 93.2, 73.1, and 62.9%. Internal validation cohort: The 1-, 3-, and 5-year OS incidence rates were 99.4, 92.4, and 84.5% in the low-risk group and 91.2, 69.0, and 56.8% in the high-risk group, respectively; the 1-, 3-, and 5-year CSS incidence rates were 99.5, 93.5, and 87.3% in the low-risk group and 93.4, 74.1, and 64.2%. For the external validation cohort: the 1-, 3-, and 5-year OS incidence rates were 100.0, 90.1, and 85.6% in the low-risk group and 90.8, 73.5, and 54.7% in the high-risk group, respectively; the 1-, 3-, and 5-year CSS incidence rates were 100.0, 94.6, and 86.5% in the low-risk group and 92.9, 72.5, and 64.0% in the high-risk group, respectively. Risk stratification can effectively avoid the survival paradox in traditional TNM staging. It can guide personalized decision making clinical patients more accurately. In addition, we analyzed the effect of different subtypes on the probability of patient survival. In the low-risk and high-risk groups, there were significant differences in OS and CSS by subtype (Supplementary Figure 2), 1-year, 3-year, and 5-year OS and CSS are shown in Supplementary Table 3. Patients with TNBC had the lowest survival rate, and patients with LuminalB had a higher survival rate than those with LuminalA.

## Discussion

Previous studies have shown that LABC has been treated for 100 years (3). Radical surgery has been considered to be the standard treatment for many types of breast cancer, including LABC. However, other preoperative systemic therapies, including neoadjuvant chemotherapy, neoadjuvant endocrine therapy, and neoadjuvant chemotherapy combined with targeted therapy are now widely used as standard treatments for LABC patients (2). Previous studies reported that nomograms can predict the survival time of patients with different stages of breast cancer (26, 27). However, there is no reliable tool for predicting OS and CSS in LABC patients.

The survival times of patients with different cancers vary significantly. Moreover, patients with the same type of cancer and TNM stage may have different survival outcomes, indicating that the TNM staging system is not an ideal prediction parameter for all cancer patients (28). Patients with positive regional lymph nodes status without T-stage intervention are classified as stage IIB, stage IIIA, and stage IIIC, whereas those with negative regional lymph nodes status, stage T3 and T4 are categorized as stage IIB and stage IIIB, respectively, based on the 7th edition of the AJCC staging system. In this study, we found that patients with stage IIIB had worse survival outcomes than those with stage IIIC, indicating that the T stage has a greater impact on LABC survival compared with the N stage. This is consistent with the rectal cancer model predictions (29). Therefore, a robust prognostic indicator or tool is needed to guide individualized treatment of LABC patients (24). In the present study, risk stratification was performed to differentiate the prognosis of LABC patients and provide a more accurate basis for decision-making regarding LABC treatment.

After Lasso regression analysis of 12 relevant clinical characteristics of LABC patients in the SEER database, nine variables were screened including age at diagnosis, marital status, grade, subtype, T-stage, N-stage, surgery, radiotherapy, and chemotherapy. It has been reported that older patients with locally advanced rectal cancer and rectal cancer have a poor prognosis (29, 30). Similarly, we found that elderly LABC patients had a poor prognosis. This may be explained by the fact that old age increases the severity of comorbidities and the risk of comorbid underlying disease, which decreases their tolerance to radiotherapy and surgery (31, 32).

Apart from the common risk factors, we found that marital status was an independent prognostic factor affecting OS and CSS in LABC patients. Previous studies have also indicated that divorced or single patients have a higher risk of cancer metastasis, under-treatment, and death (33). The effect of marital status on the prognosis of tumors has been reported in several tumors. Tan et al. analyzed 13,755 colon cancer patients from the SEER database (7,815, married and 5,940, unmarried) and found that the proportion of LARC was higher in unmarried patients than in married patients (30). Moreover, unmarried patients were less likely to undergo surgery compared with married patients. Divorced or single breast cancer patients have less psychological, social, and financial support compared with their married counterparts, which may worsen their prognosis. Therefore, such patients require appropriate psychosocial support from medical staff to improve their survival outcomes.

Studies have also shown that histologic grade, T-stage, and N-stage affect the survival prognosis of LABC patients. For instance, higher histologic grade, lower differentiation, and later T-stage and N-stage breast cancer are associated with poor survival prognosis (34, 35).

Herein, LABC patients with different subtypes had different survival outcomes. Triple-negative breast cancer showed the worst prognosis, whereas HR+ Luminal A and Luminal B exhibited the best prognosis. Endocrine therapy has been shown to improve the prognosis of HR+ patients with relatively stable disease (36). However, patients with high-risk HR+ with rapid disease progression do not benefit from endocrine therapy and are treated with symptom control-based therapy (37, 38). However, the risk level of the disease could not be assessed. This study risk-stratified LABC patients for OS showing that 27.2% (6,216/ 22,842) of hormone receptor-positive LABC patients were in the high-risk stratum and could be given conservative treatment. Moreover, 44.6% (10,185/22,842) of patients with low prognostic scores achieved long-term survival, suggesting that endocrine therapy can improve the prognosis of patients in this group. Similarly, for CSS, it was shown that 26.2% (5,979/22,842) of hormone receptor-positive LABC patients were in the high-risk stratum, and 45.6% (10,422/22,842) of patients with low prognostic scores achieved long-term survival. Endocrine therapy improves the survival of patients. Targeted drugs have been reported to improve the survival rate of HER2-positive breast cancer patients (1). Therefore, patients without HR+ and HER2 receptor-positive (triple-negative breast cancer patients) do not benefit from endocrine therapy and HER2 targeted therapy, resulting in a poor overall survival prognosis.

The constructed nomogram revealed that surgery, chemotherapy, and radiation therapy improved the survival outcomes, supporting the adoption of aggressive treatment for LABC. Previous studies have reported that neoadjuvant chemotherapy and adjuvant radiotherapy can prolong the survival of LABC patients, but not for those with decreased local control (39). We did not evaluate the impact of treatment on survival due to insufficient information on surgical and systemic treatments for LABC patients in the SEER database. However, several studies have demonstrated that surgery improves the survival prognosis of patients with locally advanced disease. For instance, Wang et al. evaluated the American Joint Committee on Cancer 8th edition prognostic stage of LABC using an appropriate Cox model and concluded that surgery, radiotherapy, and chemotherapy are independent prognostic factors for LABC patients (28). They showed that surgery significantly improved the prognosis and quality of survival of LABC patients. Zhou et al. developed and validated a preoperative nomogram of patient survival after radical surgery for locally advanced prostate cancer (40). They included surgery in the prediction model as an independent prognostic factor for patients and compared prognosis using survival curves. Results showed that surgery improved the prognosis of patients compared with those who did not undergo surgery. These retrospective studies and randomized trials suggest that primary tumor surgery may improve cancer survival by reducing the tumor load in patients. We found that patients who had surgery were at high risk compared to those who did not have surgery. In terms of type of surgery, we found that breast-conserving surgery had a lower risk score compared with mastectomy. This finding provides a treatment strategy for appropriate LABC patients, especially those with a strong desire to preserve the breast.

There are several notable advantages in this study. First, to our knowledge, this study is the first to develop and validate two nomograms for predicting OS and CSS in patients with LABC. Second, this study incorporates risk factors other than TNM stage that affect the prognosis of patients with LABC, and we have developed reliable nomograms for patients with LABC while also classifying them into high and low risk groups. The definition of risk stratification can provide a basis for prognostic judgment and individualized treatment plans to some extent. It can provide additional information for clinical work. Finally, the prediction models we developed can be used to improve the TNM staging system or as a complementary version. Because our models obtained higher C-index and AUC values compared with the conventional TNM staging, this indicates that our nomograms have better prognostic ability than TNM staging. In the DCA curve, our two nomograms exhibited a good degree of clinical benefit than TNM staging. Currently, a nomogram is known to be developed and applied to elderly LABC patients, such as Meng et al. (41), who included 10,697 elderly LABC patients from 2010 to 2017 in the SEER database and developed an OS prediction model for elderly LABC to assist in patient treatment and follow-up. With the update of the SEER database and the improvement of patient clinical information, this study included 22,842 LABC patients from 2010 to 2015, selected more patient clinical characteristics, and then constructed OS and CSS prediction models applicable to LABC patients of all ages. Most importantly, we validated the potential advantages of the model by external validation and outperformed previous studies on older LABC patients in terms of number of cases and c-index accuracy.

This study had some limitations. Firstly, SEER database has no specific information of neoadjuvant chemotherapy, treatment plan, and postoperative examination. These undescribed factors may affect the overall results. Secondly, being a retrospective study, the risk of selection bias in the construction of prediction model cannot be ruled out. In addition, important factors, such as patient's physical condition, complications, BMI, genetic data, and tobacco use, were not evaluated. Finally, BMUH has a rare number of LABC of other histological types except for patients with invasive ductal carcinoma. Therefore, external validation of other histological types cannot be performed. Despite these limitations, the results obtained showed that the constructed nomogram had high sensitivity, specificity, and prediction accuracy.

## Conclusions

This study developed two nomograms based on the SEER database to predict OS and CSS of LABC patients at 1, 3, and 5 years, and our model was found to have good accuracy, reliability and clinical applicability through internal and external validation. Compared with conventional TNM staging, our model is more advantageous in predicting survival. It can help clinicians more accurately assess the prognosis of patients and thus develop more individualized treatment plans.

## Data availability statement

The original contributions presented in the study are included in the article/Supplementary material, further inquiries can be directed to the corresponding authors.

## Author contributions

SW and FY: conception, design, development of methodology, analysis, interpretation of data, and study supervision. CH: acquisition of data. FY, SW, and CH: writing, review, and/or revision of the manuscript. FY, SW, and YZ: administrative, technical, or material support. All authors participated in the writing of the final manuscript and approved the final submission.

## Funding

This work was supported by National Natural Science Youth Fund Committee of China [No. 81902702] and National Key Research and Development Project [Nos. 2018YFC0114705 and BY2016KYQD23].

## Conflict of interest

The authors declare that the research was conducted in the absence of any commercial or financial relationships that could be construed as a potential conflict of interest.

## Publisher's note

All claims expressed in this article are solely those of the authors and do not necessarily represent those of their affiliated organizations, or those of the publisher, the editors and the reviewers. Any product that may be evaluated in this article, or claim that may be made by its manufacturer, is not guaranteed or endorsed by the publisher.

## Supplementary material

Supplementary Figure 1 LASSO regression model was used to select characteristic impact factors for OS and CSS. **(A)** Lasso coefficients for the nine features of OS; **(B)** Selection of tuning parameter (λ) for LASSO model of OS; **(C)** Lasso coefficients for the nine features of CSS; **(D)** Selection of tuning parameter (λ) for LASSO model of CSS.

Supplementary Figure 2 Kaplan-Meier curves of OS for LABC patients with different subtypes in the low- **(A)** and high-risk **(B)** groups in total set. Kaplan-Meier curves of CSS for LABC patients with different subtypes in the low- **(C)** and high-risk **(D)** groups in total set.

Supplementary Table 1 C-index for the nomograms and TNM 7th stage in patients with LABC.

Supplementary Table 2 AUC for the nomograms and TNM 7th stage in patients with LABC.

Supplementary Table 3 Survival rate of different subtypes in LABC risk stratification.

Supplementary material TRIPOD report checklist.
