# Supplementary figures and images for: Development and validation of nomograms for predicting overall survival and cancer specific survival in locally advanced breast cancer patients: A SEER population-based study
Source: Front Public Health. 2022 Sep 20;10:969030. doi: 10.3389/fpubh.2022.969030 (PMC9530359; doi:10.3389/fpubh.2022.969030)

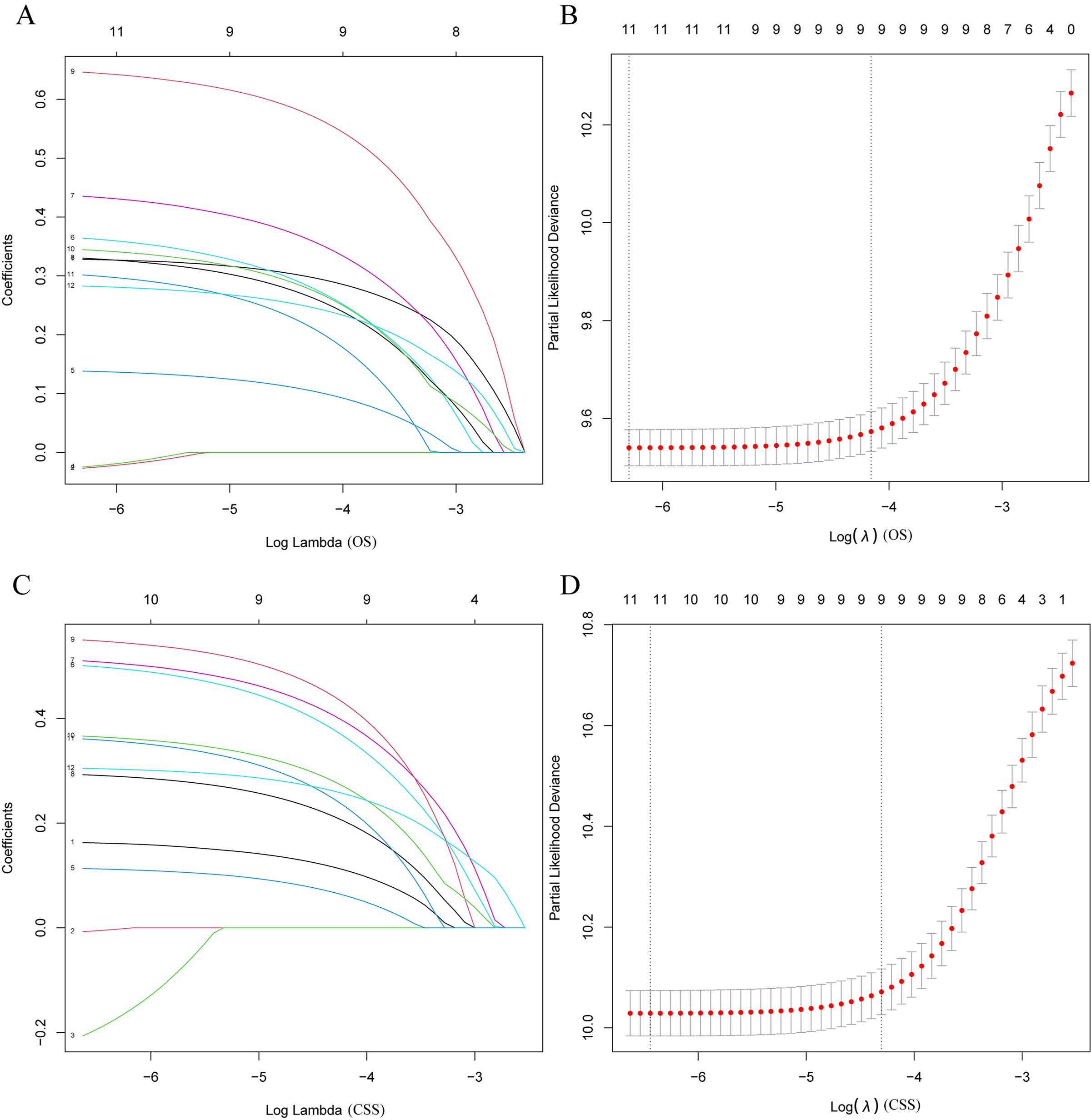

Supplement: Supplementary Figure 1 — LASSO regression model was used to select characteristic impact factors for OS and CSS. (A) Lasso coefficients for the nine features of OS; (B) Selection of tuning parameter (λ) for LASSO model of OS; (C) Lasso coefficients for the nine features of CSS; (D) Selection of tuning parameter (λ) for LASSO model of CSS. [file Image_1.TIF]

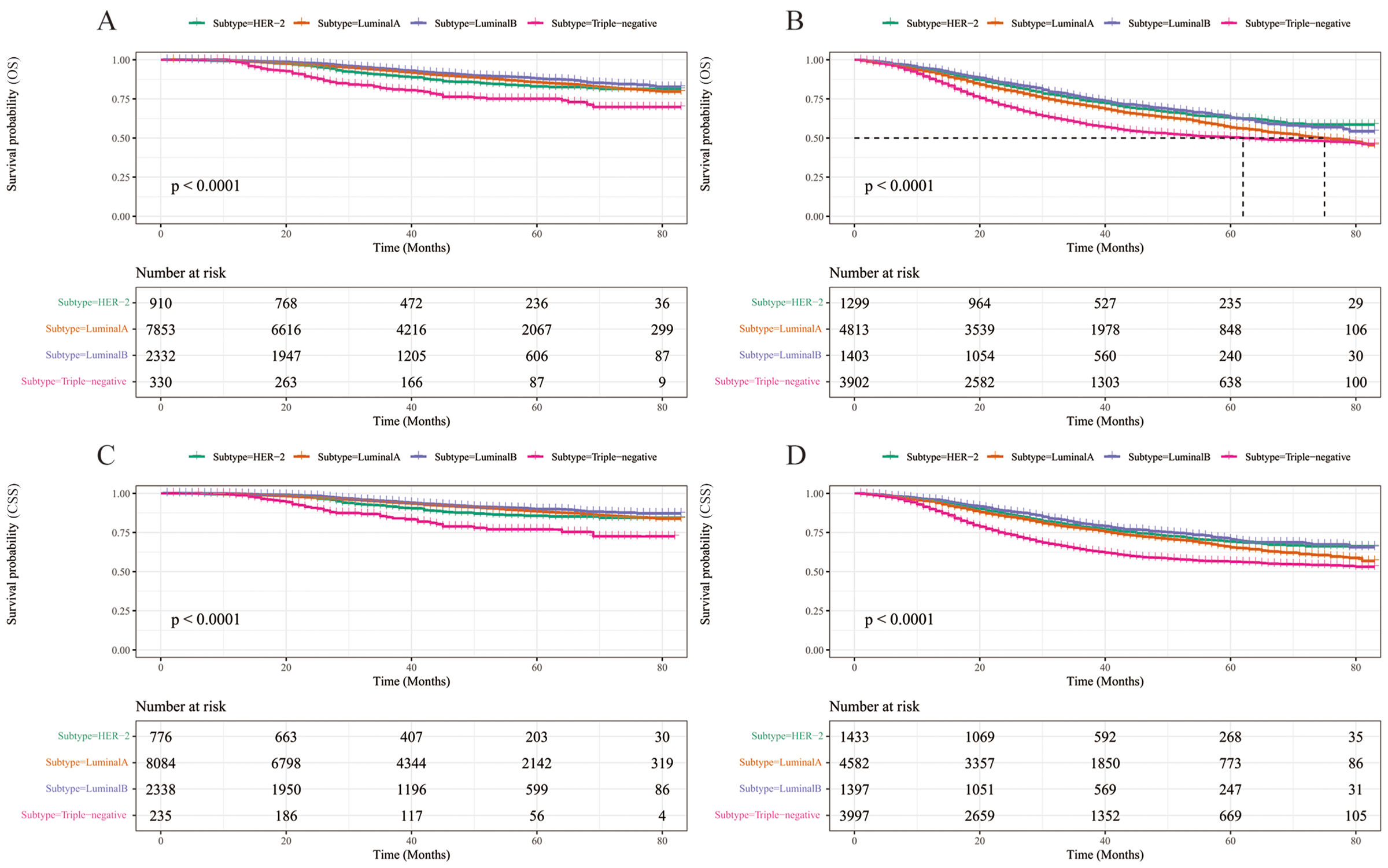

Supplement: Supplementary Figure 2 — Kaplan-Meier curves of OS for LABC patients with different subtypes in the low- (A) and high-risk (B) groups in total set. Kaplan-Meier curves of CSS for LABC patients with different subtypes in the low- (C) and high-risk (D) groups in total set. [file Image_2.TIF]
